# Safety and effectiveness of low-protein diet supplemented with ketoacids in diabetic patients with chronic kidney disease

**DOI:** 10.1186/s12882-018-0914-5

**Published:** 2018-05-09

**Authors:** Vincenzo Bellizzi, Patrizia Calella, Julia Nava Hernández, Verónica Figueroa González, Silvia Moran Lira, Serena Torraca, Rocio Urbina Arronte, Pietro Cirillo, Roberto Minutolo, Rafael A. Montúfar Cárdenas

**Affiliations:** 1 Division of Nephrology, Dialysis and Transplantation, University Hospital San Giovanni di Dio e Ruggi d’Aragona, Via San Leonardo, 84131 Salerno, Italy; 2 Department of Movement and Wellness Sciences, University of Naples Parthenope, Naples, Italy; 3 Centro de Atención Nutricional Fresenius Kabi, Ciudad de México, Mexico; 4 Division of Nephrology, University Hospital Policlinico di Bari, Bari, Italy; 5 Division of Nephrology, University of Campania, Luigi Vanvitelli, Napoli, Italy

**Keywords:** Low-protein diet, Ketoacids, CKD, Diabetes, Protein-energy wasting, Insulin resistance

## Abstract

**Background:**

The impact of the low-protein diet on nutrition in CKD diabetics is uncertain.

**Methods:**

The metabolic and nutritional effects of a low-protein (0.5–0.6 g/kg/d), normal-high energy (30–35 kcal/kg/d) diet supplemented with ketoacids (LPD-KA) were prospectively evaluated in CKD patients with (DM) and without (non-DM) diabetes mellitus.

**Results:**

197 patients on CKD stages 3–5 were enrolled. DM (*n* = 81) and non-DM (*n* = 116) were comparable for gender (Male 58 vs 55%), age (66 ± 9 vs 63 ± 18 years), renal function (eGFR 23 ± 13 vs 24 ± 13 mL/min). After 6-month, serum urea (DM: 131 ± 58 to 105 ± 49 mg/dl, *p* < 0.05; non-DM: 115 ± 52 to 88 ± 36, p < 0.05) and phosphate (DM: 4.5 ± 1.3 to 4.1 ± 1.2 mg/dl, *p* = 0.06; non-DM: 4.3 ± 1.0 to 3.7 ± 0.8, p < 0.05) declined. Fasting glucose decreased in DM (126 ± 52 to 103 ± 29 mg/dl, p < 0.05) without insulin dose increase. These effects were preserved after 3-year. Serum albumin did not change after 6 months (DM: 3.7 ± 0.6 to 3.8 ± 0.4 mg/dl; non-DM: 4.0 ± 0.6 to 4.0 ± 0.4) and in the long-term. Body weight (BW) declined after the diet start (DM: 68.9 ± 14.3 to 65.1 ± 12.1 kg, *p* < 0.05; non-DM: 66.6 ± 15.1 to 64.1 ± 15.1, *p* < 0.05) and was stable at 6 months and 3 years. Muscle strength at baseline was reduced in all patients and remained stable during the diet period. Changes of nutritional markers during the study were similar among groups and diabetes was not associated to any nutritional change at the multivariate analysis. As attain wasting, lower BMI (< 23 kg/m^2^) and albumin (< 3.8 g/dl) levels were present in 1/3 patients at start and along 3 years, cholesterol never dropped below the lower threshold (< 100 mg/dl) and poorer FM (< 10%) was less than 10% during the study in both groups.

**Conclusions:**

In diabetic CKD patients a low-protein diet supplemented with ketoacids improves uremia and diabetes, causes sudden decline of body weight which remains stable over time and has not a negative effect on wasting and muscle mass and fitness. In diabetic CKD patients the LPD-KA is safe and the nutritional impact is the same as in non-diabetics CKD.

## Background

During the last decades the prevalence of chronic kidney disease (CKD) increased worldwide, the end-stage renal disease (ESRD) has become one of the major causes of mortality in the world and the use of renal replacement therapy is projected to double by 2030 [1, 2]. Additionally, it emerged that dialysis is not capable to prolong life while, in contrast, reduces the quality of the life and the individual functional capacity in most patients [3]. Therefore, in addition to a population-based strategies to prevent ESRD, an efficacious conservative treatment for non-dialysis CKD patients is mandatory. The nutritional treatment is a main part of the conservative treatment for non-dialysis CKD, aiming to reduce the metabolic disturbance of CKD, the signs and symptoms of uremia and the progression of renal disease and may delay dialysis [4]. Consequently, the low-protein diet, which is the cornerstone of the nutritional treatment for CKD, plays a central role in the conservative management of CKD [5].

A major possible drawback which has been related to the use of the low-protein diet in CKD is the risk of protein-energy wasting (PEW) that is the loss of body store of proteins and energy reserves. The cause of PEW in renal patients is multifactorial and a reduced nutrient intakes may contribute to the syndrome in addition to several conditions such as inflammation or resistance to insulin which play a crucial role in the loss of muscle mass [6]. The most risky nutrient deprivation for CKD patients is the energy intake, rather than the protein intake [7]. Indeed, PEW is very rare in CKD patients on regular low-protein diet if adequate energy intake is observed and when the dietary regimen goes on under a regular counselling and a close nutritional monitoring [8–10]. Among the low-protein diets for CKD, there is a very restricted one which is supplemented with aminoacids and ketoacids (LPD-KA) in order to cover the minimum body nitrogen need and to minimalize the nitrogen load. The LPD-KA has been proven to achieve a more rigorous metabolic control, a reduction of the cardiovascular risk factors related to advanced CKD and to further slow-down the CKD progression delaying the start of dialysis [11, 12]. In contrast, a very restricted renal diet may expose the CKD patient to higher risk of insufficient nutrient intake and of PEW. A regular clinical follow-up and a strict dietitian assistance, however, allows to maintain of a good nutritional status with such LPD-KA, even in elderly patients [13].

Diabetes mellitus (DM) is rapidly increasing all over the world and diabetic nephropathy is estimated to develop in four out of ten DM patients, representing the main cause of CKD; indeed, around one over three CKD patients is affected by diabetes [14]. Therefore, a low-protein diet supplemented with ketoacids could be a resource for diabetic CKD patients, but these subjects are more inflamed, resistant to insulin, comorbid, hyper-catabolic and, at least in theory, they might require more proteins and aminoacids to compensate the catabolic status [15]. Hence, a restricted diet such as the low-protein diet – LPD may not provide sufficient amounts of nutrients and energy and, as a result, could increase the risk of PEW. Furthermore, because of the reduced energy amount resulting from the lower protein intake, the LPD contains more carbohydrates to reach the energy request and the high carbohydrates can worsen the control of diabetes. Safety data on the relation between long-term use of protein restriction and PEW in diabetic CKD are scarce. This observational, prospective study aimed at evaluating the effects of a LPD supplemented with ketoacids on long-term nutritional outcome in diabetic CKD patients.

## Methods

### Patients and groups

This is a observational, prospective study conducted in the renal clinic of the Centro de Atención Nutricional Fresenius Kabi in México City, Mexico. Consecutive outpatients with non-dialysis chronic kidney disease (CKD) on regular nephrology and nutritional follow-up were enrolled in the period between January 1st 2010 and August 1st 2013; the end of follow-up was on August 31st 2015. The research protocol involving these patients was approved by the local ethical committee, the “Comité Local de Investigación y Ética del Hospital de Especialidades Centro Médico Nacional la Raza del Instituto Mexicano del Seguro Social”, and all subjects enrolled into the study gave their informed consent to participate to the research.

Selection criteria were: a) age ≥ 18 years; b) CKD stage 3 or higher by estimated GFR; c) follow-up of at least 6 months in CKD clinic; d); stable renal function (variation of eGFR < 30% in the last 3 months). Exclusion criteria were: a) previous consumption of LPD-KA; b) history of dialysis or renal transplantation; c) current treatment with immunosuppressive drugs; d) pregnancy or lactation; e) malignancy; f) congestive heart failure (NYHA class III-IV); g) severe undernutrition (body mass index – BMI < 20 kg/m^2^ and serum albumin < 3.0 g/dl, or BMI < 17.5 kg/m^2^ irrespective of the albumin value, or unintentional body weight – BW reduction > 7.5% in the last 3 months); h) inability of spontaneous feeding; i) any acute illness during the previous 3 months.

### Design of the Study and Nutritional Treatment

Selected patients were monitored for a run-in period prior to the final enrolment. If the inclusion criteria were confirmed, patients underwent a dietitian evaluation and were prescribed a low-protein diet supplemented with ketoacids (LPD-KA), containing 0.5–0.6 g proteins/kg iBW/day, low amount of purines, sodium (< 2 g/day) and phosphorus (< 800 mg/day) and normal-high amount of energy (30–35 kcal/kg iBW/day). The diet was supplemented with a mixture of aminoacids and ketoacids (Ketosteril®, Fresenius Kabi, 1 tab/ 5–7 kg BW/day) and omega-3 fatty acids (3 g/day) were recommended.

The dietary plan was explained to patients during a nutritional consultation, using several educational tools like examples of different kind of meals, recipes and food models. In addition, a list of food equivalents in which foods are grouped by the content of proteins, potassium and energy, according to the Mexican Food Equivalent System – MFES [16], was provided to patients. The nutritional treatment was under the supervision and the continuous support of dietitians and each patient has been guided to create his own menu.

The study lasted at least 6 months; all patients along this period were evaluated at baseline, and after the start of the diet at 1, 2 and 6 month-time (short-term observation). At this point, patients were asked to continue the diet for at least two years, with further clinical evaluations every 6 months (long-term observation). At each visit, blood samples were collected to determine the laboratory data and clinical and nutritional evaluations were performed by a nephrologist and a dietitian. The outcome of the study was the nutritional status.

### Measurements and calculations

In the morning, after a fasting period of 10 to 12 h, patients underwent blood sampling. Serum creatinine, electrolytes, glucose, urea, uric acid, haemoglobin, proteins, albumin and lipids were analysed by standard methods. Renal function was estimated with the GFR calculated by the CKD-EPI equation and the CKD stages were defined according to KDIGO guidelines [17, 18].

At each visit, a clinical evaluation, including body composition and muscle strength measurements, was done. Body weight was measured to the nearest 0.1 kg and height to the nearest 0.5 cm. BMI was calculated as the ratio weight/height^2^ (kg/m^2^). Waist and hip circumferences were also measured and the W/H ratio was calculated.

The body composition was estimated by bioimpedance analysis – BIA, using a multi-frequency Body Composition Analyzer (PLUSAVIS 333; frequencies range: 5–50-250 kHz; Jawon medical Co. Ltd.; Korea). The measures were taken with patient wearing only a gown. The electrodes were placed on the feet and both hands. The subject had to be in the post-absorptive state and was placed in standardized conditions: quiet environment, ambient temperature of 22–24 °C [19, 20]. The evaluated BIA derived variables were total body water (TBW) and fat-free mass (FFM); fat mass (FM) was calculated as the difference between BW and FFM.

The muscle strength was measured by a digital hand grip dynamometer (*T.K.K.* 540.1 *GRIP D*; Takei Scientific Instruments Co. Ltd.; Niigata, Japan). The measures were taken on the dominant hand. The patient was standing upright; he/she was asked to hold the device with the grip meter indicator facing outward, to leave the arm down naturally and finally to clasp the grip with full force for 3 s. Three measures were taken within a half-minute of each other and the mean value was reported.

### Statistics

Statistical analyses were performed using the statistical software SPSS version 22 (IBM SPSS Statistics). Continuous variables were expressed as mean ± standard deviation (SD) and categorical variables were expressed as percent. During the analysis, the patients were grouped in diabetics (DM) and without diabetes (non-DM). Comparison of all parameters at baseline and at the end of follow-up periods and comparison of parameters changes during the study between groups were performed by unpaired Student *t*-test. Comparison within group was made by paired Student *t*-test, ANOVA or *chi-square*-test as appropriate. To verify if diabetes was associated with changes during the long-term study of main clinical and nutritional parameters (body weight, blood pressure, muscle strength, serum levels of urea, uric acid, phosphates and albumin), we performed multiple regression analyses. For each parameter separately, we built a multivariable model including the variation during the follow-up (final-basal delta) adjusted for the same parameter at baseline, diabetes and potential confounders, i.e. the individual characteristics differing between groups at baseline (gender, age and eGFR). A two-tail *P* value < 0.05 was considered statistically significant.

## Results

### Patients

Among the screened patients, those who refused the nutritional treatment or declared they cannot be adherent to the LPD or did not meet the inclusion criteria were excluded; these patients were not submitted to any further evaluation.

Two hundred nine patients were selected on the basis of the initial inclusion criteria; after the run-in period, 197 patients (DM = 81; non-DM = 116) were enrolled into the study, starting the low-protein diet supplemented with ketoacids – LPD-KA. All patients completed the 6 months diet period (short-term observation). Afterwards, 86 patients did not continue the LPD-KA; among the remaining 111 patients who continued the diet, 47 did not reach a minimum follow-up of 24 months at the end of the study. In summary, 64 patients (DM = 29; non-DM = 35) remained on the LPD-KA for 2 years or more, with a mean follow-up of 38 months (long-term observation).

### Short-term observation

In the enrolled patients the mean age was 64 years and males were 57%. Renal diseases was type-1 = 6% and type-2 = 94% in diabetes mellitus (DM) and hypertension = 59%, glomerular nephritis = 3%, interstitial nephritis = 8%, other/unknown = 31% in non-DM groups. The mean renal function measured by eGFR was 23,5 ml/min with 25, 43 and 32% of patients in CKD stages 3, 4 and 5, respectively. Except for renal disease, there were no differences between groups (Table 1). The patients excluded from the study after the run-in period had similar characteristics.

**Table 1:** Individual characteristics of CKD patients on low-protein diet and ketoacids followed in the short-term (6 months)

	Total	Diabetes	non-DM
N	197	81	116
Age, *years*	64,1 ± 15,3	65,7 ± 9,3	62,9 ± 18,3
Gender, *%M*	57	58	55
Height, *mt*	1,60 ± 0,10	1,60 ± 0,09	1,59 ± 0,10
Renal disease, *%*			
Diabetes type 1	2,5	6,2^*^	–
Diabetes type 2	38,6	93,8^*^	–
Hypertension	34,5	–	58,6
Glomerular nephritis	1,5	–	2,6
Interstitial Tubular Nephritis	4,6	–	7,8
Other disease	7,1	–	12.0
Unknown	11,2	–	19.0
sCreatinine, *mg/dl*	3,38 ± 1,84	3,57 ± 2,09	3,25 ± 1,64
eGFR, *ml/min*	23,5 ± 12,9	22,7 ± 13,0	24,01 ± 13,0
CKD stage, *%*			
3	25.0	24,7	25,2
4	43,4	39,5	46,1
5	31,6	35,8	28,7
Ketoacids dose, *pill/day*	8,4 ± 2,8	8,8 ± 2,8	8,1 ± 2,8

*non-DM* non Diabetes Mellitus, *s* serum, *eGFR* estimated glomerular filtration rate, *CKD* chronic kidney disease

^*^p < 0.05 vs. non-DM

Dietary treatment produced several significant metabolic changes in all patients (Table 2). As expected, serum urea, which was a little different among groups at baseline (*p* < 0.05), significantly decreased by the same extent (*p* < 0.05) in both DM and non-DM. Uric acid level decreased in all patients, but significantly only in non-DM (*p* < 0.05), with a final similar level between groups. Serum phosphate decreased in non-DM (*p* < 0.05), with a final lower level as compared to DM (*p* < 0.05). The fasting glucose in DM was always significantly higher versus non-DM (*p* < 0.05) and significantly declined in DM (*p* < 0.05), with no increase in the prescription of insulin dose.

**Table 2:** Clinical and laboratory follow-up of CKD patients on low-protein diet and ketoacids followed in the short-term (6 months)

	Diabetes	(*n* = 81)	non-DM	(*n* = 116)
	basal	final	basal	final
SBP, *mmHg*	137 ± 20^*^	130 ± 21	127 ± 15	129 ± 16
DBP, *mmHg*	78 ± 18	74 ± 9	76 ± 8	76 ± 10
Urea, *mg/dl*	131 ± 58^*^	105 ± 49^*#^	115 ± 52	88 ± 36^#^
Uric Acid, *mg/dl*	7,0 ± 2,1	6,4 ± 1,8	7,4 ± 3,2	6,2 ± 1,9^#^
Glucose, *mg/dl*	126 ± 52^*^	103 ± 29^*#^	97 ± 18	97 ± 25
Sodium, *mEq/dl*	139 ± 4	140 ± 4	139 ± 4	139 ± 4
Potassium, *mEq/dl*	5,0 ± 0,8	4,7 ± 0,6	4,8 ± 0,7	4,7 ± 0,6
Calcium, *mg/dl*	9,1 ± 0,8	9,1 ± 0,6	9,3 ± 1,5	9,3 ± 0,6
Phosphate, *mg/dl*	4,5 ± 1,3	4,1 ± 1,2^§*^	4,3 ± 1,0	3,7 ± 0,8^#^
Hemoglobin, *g/dl*	12,0 ± 1,7	11,7 ± 1,5	12,2 ± 2,2	12,3 ± 1,7
Total proteins, *g/dl*	6,7 ± 0,7	6,8 ± 0,5	6,9 ± 0,9	7,0 ± 0,4

*non-DM* non Diabetes Mellitus, *SBP* systolic blood pressure, *DBP* diastolic blood pressure

^#^*p* < 0.05 vs. basal; ^§^*p* = 0.06 vs. basal; ^*^p < 0.05 vs. non-DM

**Fig. 1:**
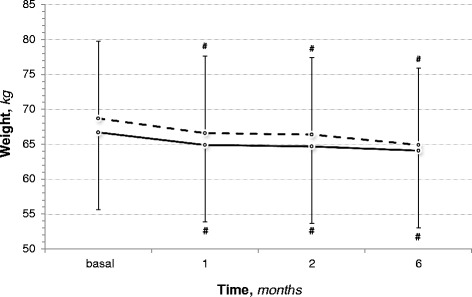
Body weight changes in CKD patients on low-protein diet and ketoacids during a short-term period (6 months); −---- = Diabetes Mellitus; ^_____^ = non Diabetes Mellitus

Nutritional status and body composition, evaluated by the combination of several parameters of common clinical use, were similar among groups at the start and did not worse after the intervention (Table 3). Serum albumin, which was reduced in DM patients versus non-DM at baseline (*p* < 0.05), did not change. Serum cholesterol slightly decreased in either DM or non-DM. Triglycerides and lymphocytes did not change. Body weight soon after the first month of diet significantly declined in both groups (p < 0.05) (Fig. 1); afterwards, it remained stable until the last observation (*p* < 0.05 vs. basal; NS vs. 1 and 2 months). The body weight decline was associated with a slight reduction of absolute body water (DM: NS; non-DM: *p* < 0.05), fat mass (DM and non-DM: *p* < 0.05) and fat free mass (DM: NS; non-DM: *p* < 0.05). Fractional fat free mass did not lower in both groups. No differences between groups were detected for either the extent of changes (final-basal delta) or the final observation values.

**Table 3:** Nutritional follow-up of CKD patients on low-protein diet and ketoacids followed in the short-term (6 months)

	Diabetes	(*n* = 81)	non-DM	(*n* = 116)
	basal	final	basal	final
BMI, *kg/mt*^*2*^	26,7 ± 5,8	24,2 ± 6,4	26,0 ± 4,5	25,0 ± 4,9^#^
Albumin, *g/dl*	3,7 ± 0,6^*^	3,8 ± 0,4	4,0 ± 0,6	4,0 ± 0,4
Cholesterol, m*g/dl*	186 ± 50	165 ± 37^#^	177 ± 47	166 ± 38^#^
Tryglicerides, m*g/dl*	183 ± 86	167 ± 83	171 ± 88	165 ± 78
Lynfocites, *n*	2.304 ± 2.263^*^	1.640 ± 523^*^	2.210 ± 2.605	1.782 ± 753
W/H ratio	0,91 ± 0,08	0,89 ± 0,08	0,92 ± 0,08	0,91 ± 0,08^#^
TBW, *Liters*	36,0 ± 7,4	35,4 ± 7,6	33,8 ± 8,0	32,7 ± 8,1^#^
TBW, *%bw*	50 ± 13	53 ± 10	51 ± 8	51 ± 6
FM, *Kg*	18,6 ± 8,5	16,6 ± 7,1^#^	19,4 ± 7,4	18,4 ± 6,8^#^
FM, *% bw*	27 ± 9	25 ± 9^*^	29 ± 8	29 ± 7
FFM, *Kg*	49,9 ± 10,2	48,9 ± 9,9	47,1 ± 11,0	45,5 ± 11,3^#^
FFM, *% bw*	73 ± 9	75 ± 9^*^	71 ± 8	71 ± 7
BMR, *Kcal*	1.167 ± 167	1.171 ± 163^*^	1.159 ± 209	1.092 ± 195^#^
Muscle strength, *kg*	21,7 ± 8,1	23,9 ± 7,5^#^	24,8 ± 8,7	23,9 ± 7,9
Male	25,4 ± 7,6^°^	27,7 ± 5,4	29,2 ± 8,2	27,7 ± 7,8
Female	15,9 ± 5,6^°^	16,9 ± 5,2	19,0 ± 5,3	20,0 ± 5,9
Muscle strength changes, *Δ*	–	2,2 ± 3,3	–	-0,9 ± 4,1

*non-DM* non Diabetes Mellitus, *BMI* body mass index, *W/H* waist/hip, *TBW* total body water, *FM* fat mass, *FFM* fat free mass, *BMR* basal metabolic rate

^#^*p* < 0.05 vs. basal; ^*^p < 0.05 vs. non-DM;^°^*p* = 0.06 vs. non-DM

According to hand-grip references (lower threshold: 30 and 20 kg for Male and Female, respectively [21]), muscle fitness was poor in all patients and even more reduced in diabetics; the differences among diabetics and non-DM were similar for gender sub-groups (Table 3). During the low protein diet, muscle strength increased in DM but not in non-DM group; specifically, it was observed a slight clinical trend to decline in non-diabetic Males (around 5%) and to increase in all Females (around 5%) and diabetic Males (around 8%), with similar final levels for either the whole DM and NON-DM groups or the male sub-groups (Table 3).

### Long-term observation

64 patients (DM = 29; non-DM = 35) on LPD-KA were studied for a period longer than 2 years (38 ± 13 months). Diabetic group was a little older and had a slight lower renal function whit more patients in CKD stage 5; these differences, however, were not significant (Table 4).

**Table 4:** Basal characteristics of CKD patients on low-protein diet and ketoacids followed in the long-term (at least 2 years)

	Total	Diabetes	non-DM
N	64	29	35
Age, *years*	65,8 ± 14,7	68,6 ± 7,6	63,5 ± 18,4
Gender, *%M*	68,8	65,5	71,4
Height, *mt*	1,60 ± 0,09	1,60 ± 0,09	1,59 ± 0,09
Renal disease, *%*			
Diabetes type 1	1,6	3,4^*^	–
Diabetes type 2	43,8	96,6^*^	–
Hypertension	40,6	–	74,3
Glomerular nephritis	1,6	–	2,9
Tubulo-interstitial nephritis	4,7	–	8,6
Other disease	1,6	–	2,9
Unknown	6,3	–	11,4
sCreatinine, *mg/dl*	3,02 ± 1,51	3,43 ± 1,90	2,68 ± 1,00
eGFR, *ml/min*	25,8 ± 12,7	23,3 ± 12,7	27,9 ± 12,4
CKD stage, *%*			
3	31,2	24,1	37,1
4	46,9	44,8	48,6
5	21,9	31	14,3
Ketoacids dose, *pill/day*	8,3 ± 2,7	8,6 ± 2,8	8,1 ± 2,6
Ketoacids duration, *months*	37,7 ± 13,4	34,8 ± 12,8	40,0 ± 13,7

*non-DM* non Diabetes Mellitus, *s* serum, *eGFR* estimated glomerular filtration rate, *CKD* chronic kidney disease

^*^p < 0.05 vs. non-DM

During the long-term follow-up the LPD-KA had similar metabolic effects than in the short term (Table 5). Of relevance, serum urea and phosphates decreased (*p* < 0.05) only in DM were the basal levels were higher. Uric acid markedly decreased only in DM (*p* < 0.05). Also during the long-term the fasting glucose significantly decreased in DM (*p* < 0.05).

**Table 5:** Clinical and laboratory follow-up of CKD patients on low-protein diet and ketoacids followed in the long-term (at least 2 years)

	Diabetes	(n = 29)	non-DM	(n = 35)
	basal	final	basal	final
SBP, *mmHg*	140 ± 23^*^	135 ± 20^*^	128 ± 17	127 ± 13
DBP, *mmHg*	78 ± 14	75 ± 11	73 ± 10	76 ± 10
Urea, *mg/dl*	130 ± 64^*^	108 ± 51^*^	98 ± 48	90 ± 52
Uric Acid, *mg/dl*	7,0 ± 1,7	5,8 ± 1,7^#^	6,6 ± 2,0	6,3 ± 1,7
Glucose, *mg/dl*	114 ± 55	104 ± 18^*^	99 ± 16	94 ± 15
Phosphate, *mg/dl*	4,3 ± 0,8^*^	3,8 ± 0,5^#^	3,8 ± 0,8	3,6 ± 0,7
Hemoglobin, *g/dl*	12,1 ± 1,9	12,1 ± 2,2	13,0 ± 2,3	13,0 ± 2,0
Total proteins, *g/dl*	7,0 ± 0,6	7,1 ± 0,7^*^	7,1 ± 0,6	6,7 ± 0,5^#^

*non-DM* non Diabetes Mellitus, *SBP* systolic blood pressure, *DBP* diastolic blood pressure

^#^*p* < 0.05 vs. basal; ^*^p < 0.05 vs. non-DM

**Fig. 2:**
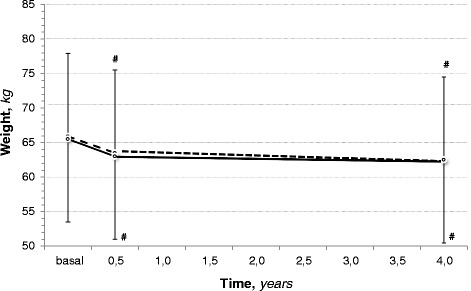
Body weight changes in CKD patients on low-protein diet and ketoacids during a long-term period (3 years); −---- = Diabetes Mellitus; ^_____^ = non Diabetes Mellitus

The same effects on the nutritional status were maintained also during the long-term LPD-KA. Albumins was almost normal and stable (Table 6). Body weight (Fig. 2) significantly (*p* < 0.05) decreased at start and then remained stable along the follow-up, with the same trend than in the short term; the body weight decline was associated with absolute fat mass slight reduction (DM: NS; non-DM: *p* < 0.05) as in the short observation. Fat free mass was unchanged.

**Table 6:** Nutritional follow-up of CKD patients on low-protein diet and ketoacids followed in the long-term (at least 2 years)

	Diabetes	(*n* = 29)	non-DM	(*n* = 35)
	basal	final	basal	final
BMI, *kg/mt*^*2*^	25,5 ± 3,3	24,3 ± 2,5^#^	25,6 ± 3,8	24,3 ± 2,9^#^
Albumin, *g/dl*	3,8 ± 0,5	3,9 ± 0,4	4,0 ± 0,4	3,9 ± 0,5
Cholesterol, m*g/dl*	173 ± 34	164 ± 23^*^	170 ± 51	165 ± 33
Tryglicerides, m*g/dl*	168 ± 85	147 ± 52	176 ± 95	155 ± 85
W/H ratio	0,90 ± 0,08	0,90 ± 0,07	0,93 ± 0,09	0,92 ± 0,09
FM, *Kg*	17,6 ± 6,4	15,9 ± 6.0	18,4 ± 6,2	16,5 ± 4,9^#^
FM, *% bw*	27 ± 9	26 ± 9	28 ± 8	27 ± 8
FFM, *Kg*	47,9 ± 9,8	46,5 ± 9,7	47,2 ± 11,9	45,7 ± 10,1
FFM, *% bw*	73 ± 8	74 ± 11	72 ± 7	72 ± 7
Muscle strength, *kg*	20,8 ± 7,9^*^	21,2 ± 7,2^*^	25,0 ± 7,8	25,5 ± 7,8
Male	24,3 ± 7,3	24,1 ± 6,9^°^	27,8 ± 6,9	28,3 ± 7,1
Female	14,1 ± 3,3^°^	15,6 ± 3,4	18,2 ± 5,5	18,6 ± 4,8
Muscle strength changes, *Δ*	–	0,3 ± 3,2	–	0,5 ± 3,6

*non-DM* non Diabetes Mellitus, *BMI* body mass index, *W/H* waist/hip ratio, *FM* fat mass, *FFM* fat free mass

^#^*p* < 0.05 vs. basal; ^*^p < 0.05 vs. non-DM; ^°^*p* = 0.06 vs. non-DM

During the long-term LPD-KA diet, muscle strength was similar to the short-term observation, with lower values in diabetics and comparable differences among groups and gender sub-groups; along the long-term follow-up hand grip remained substantially unchanged (Table 6). Overall, at baseline muscle strength was under the threshold for sarcopenia [21] in 83 and 69% DM and non-DM patients, respectively; during the study it slightly improved, being respectively 79 and 54% at the final observation.

The percent changes from baseline of the clinical and nutritional parameters during the long-term follow-up were not different among groups: body weight: − 3.9 ± 7.6 vs. -4.4 ± 8.5%; blood pressure: − 2.1 ± 15.3 vs. − 0.2 ± 12.2; muscle strength: 4.8 ± 20.0 vs. 3.9 ± 18.5; urea: − 2.8 ± 58.5 vs. -1.8 ± 40.0; uric acid: − 11.0 ± 35.7 vs. -6.1 ± 55.8; phosphates: − 7.7 ± 18.5 vs. -3.3 ± 19.8; albumin: 1.9 ± 13.4 vs. -2.0 ± 10.7; respectively for DM and non-DM (all p = NS).

Multiple regression analyses disclosed that gender, age eGFR and diabetes did not predict the changes of nutritional and clinical parameters during the study. Indeed, diabetic status was not significantly associated with changes of body weight (*P* = 0.636), blood pressure (*P* = 0.291), muscle strength (*P* = 0.416) and serum levels of urea (*P* = 0.273), uric acid (*P* = 0.229), phosphates (*P* = 0.568) and albumin (*P* = 0.243).

### Protein-energy wasting

Adherence to diet prescription is usually low in the clinical practice [22]. Indirect data strongly suggest that in this study the dietary intakes were adequate and, specifically, the protein intake lowered, as testified by the reduced serum urea levels, and the energy intake remained constant, as the body weight was unchanged along the long-term follow-up.

To obtain a comprehensive evaluation of protein-energy wasting, we applied the integrated algorithm proposed by the International Society of Renal Nutrition and Metabolism which includes four different categories of nutritional criteria with the aim to early identify the nutritional risk and make timely diagnosis of PEW in CKD (Table 7) [6]. The prevalence of both low BMI (< 23 kg/m^2^) and reduced serum albumin (< 3.8 g/dl) was around one over three patients in both groups at baseline and it was similar along the study for both parameters. Low serum cholesterol (< 100 mg/dl) was virtually absent along the entire study period. The prevalence of low fat mass (< 10% Body Weight) was very low at baseline and was stable in DM and non-DM groups during the study.

**Table 7:** Comprehensive protein-energy wasting – PEW, by mean of prevalence of major nutritional parameters, during the follow-up in CKD patients on low-protein diet and ketoacids

	Groups	basal	6 months	38 months
BMI < 23 kg/m^2^, *%*	DM	30	33	30
	non-DM	23	29	37
Albumin < 3.8 g/dl, *%*	DM	41	38	38
	non-DM	23	29	37
Cholesterol < 100 mg/dl, *%*	DM	3	0	0
	non-DM	0	0	0
Fat Mass < 10% weight*, %*	DM	7	7	3
	non-DM	3	3	3

*BMI* body mass index, *DM* Diabetes Mellitus

## Discussion

This study provides evidence that the low-protein diet supplemented with ketoacids in CKD patients with diabetes is nutritionally safe. In a cohort of well-nourished, adult outpatients with moderate to advanced CKD on regular nephrology care, a low-protein diet improved the metabolic control of uremia and diabetes. Body weight declined soon after starting the low-protein diet intervention but subsequently it remained stable along time and, importantly, muscle fitness was stable or even improved over time; no further nutritional abnormalities or body composition changes were observed and no differences between diabetics and non-diabetics occurred. Overall, in diabetic patients with CKD a low-protein diet supplemented with ketoacids improves the broad metabolic profile and does not worsen nutritional status and body composition.

The cohort in this study had similar characteristics to general CKD population [23], and diabetic and non-diabetic patients were comparable for individual, renal and nutritional characteristics, though diabetics were heavier and had a slightly reduced muscle strength at baseline. As expected by reducing protein intake in CKD, after the initiation of the low-protein diet the serum levels of urea and phosphate lowered in all patients. In diabetic subjects also fasting glucose declined soon after the start of the low-protein diet and persisted close to normal levels during the long-term follow-up on LPD-KA; no information on glycated haemoglobin is available in this study. Glucose homeostasis in CKD diabetic patients during low-protein diet has never been systematically explored and few prospective data on glycaemic control during LPDs are known. In a small prospective trial, sixty-nine diabetic patients with CKD stage 3–4, were randomized to receive a low-protein diet or a free diet; during one year of follow-up on LPD, the serum glucose level was unchanged, but the levels of glycated haemoglobin decreased [24]. Glycated haemoglobin decreased also during a low-protein diet in a RCT in eighty-two patients with progressive CKD and type-I diabetes [25]. A systematic review and meta-analysis of randomised controlled trials evaluating the effect of low-protein diet on outcomes in patients with diabetic nephropathy showed a slight but significant decrease of glycated haemoglobin while on a low-protein diet [26]. In these latter studies a low-protein diet was prescribed but not ketoacids.

The low-protein diet has a higher content of carbohydrates to counterbalance the lower energy amount resulting from the reduced dietary protein content and to reach adequate energy intake [4]. Furthermore, the glucose metabolism in CKD is impaired because of reduced insulin sensitivity [27]. Hence, the higher dietary carbohydrates and the glucose homeostasis deficiency together may lead to the worsening of diabetes control in CKD. On the other hand, the low-protein intake increases the insulin-mediated glucose removal; indeed, in CKD patients the post-absorptive plasma levels of both glucose and insulin decreased after a very-low protein (high carbohydrates) diet supplemented with ketoacids, indicating the restoring of insulin sensitivity and the improving of glucose tolerance [28]. A possible mechanism has been suggested by a recent experimental study showing that the defective insulin secretion in CKD is related to the plasma urea level, demonstrating that circulating urea directly impairs insulin secretion by pancreatic islets [29].

In this study, around 80% of diabetic patient needed less insulin doses during LPD, but it is not known how many units were actually reduced; however, data on insulin use are not consistent to support the hypothesis of an improvement of insulin resistance with the LPD-KA. Despite that, the uric acid behaviour during the study allows to speculate on improvement of insulin resistance. After six months of low-protein diet, uric acid levels decreased in all patients with a final level similar between groups; it is plausible that uric acid drop during the short time period might be dependent on the low amount of purines in the LPD-KA diet. On the other hand, in the long term the uric acid markedly decreased in diabetics. Insulin resistance and uric acid are independently linked and there is strong epidemiological evidence that hyperuricemia may be either associated to or even predict the onset of insulin resistance [30, 31]. Formerly, hyperuricemia was considered as a consequence of insulin resistance due to the reduction of urinary uric acid excretion but later it was also suggested uric acid has a contributory causal role in diabetes [31, 32]. Indeed, insulin resistance in models of metabolic syndrome can be improved by lowering serum uric acid [33, 34]. Uric acid also blocks the insulin mediated endothelial nitric oxide release that is critical for insulin action [35]. The evidences of lowering uric acid on insulin resistance in human studies are limited. However, insulin resistance has been reported to be improved by urate lowering therapy; one study reported the improvement of glycated hemoglobin levels in diabetic subjects treated with allopurinol [36]. Therefore, in our CKD diabetic patients the low-protein diet directly lowered the uric acid level and, likely, the lower urea levels obtained with the LPD-KA improved the insulin resistance which further contributed to the lowering of uric acid. Overall, the reduction of uric acid improved the glucose metabolic profile most likely throughout amelioration of insulin resistance.

With respect the impact of low-protein diet on nutritional status, diabetic CKD patients in addition to insulin resistance, are more inflamed, have a worst control of metabolic acidosis; overall, this is an hyper-catabolic condition which can accelerate protein degradation and a reduced protein intake may not satisfy the nitrogen need to counterbalance the catabolic status [15]. At least theoretically, diabetic patients require more protein and aminoacids and a low-protein diet could expose the patient to higher risk of protein-energy wasting (PEW). Therefore, when prescribing a LPD in diabetics CKD, careful attention to adequate nutrient intake and to appropriate nutritional status is mandatory. In this study, all patients received a diet containing low proteins, around the minimum threshold for a neutral nitrogen balance and the diet was supplemented with essential aminoacids and ketoacids to reach the Recommended Dietary Allowance for proteins (0.8 g per kilo weight per day) for the adult population and considered the very minimum level for diabetics with CKD [37, 38]; a normal-high amount of calories was provided to be sure to fully cover the energy need. Besides, the dietary plan was personalized for each patient by a skilled dietitian who trained the patients at start and during the diet treatment and closely monitored the nutritional status.

Serum albumin was slightly lower in diabetics at baseline and did not change in both groups during the diet treatment. In contrast, body weight declined soon after the diet start and then remained stable even in the long-term follow-up; this is not unusual after the start of a low-protein diet. For instance, in the MDRD study which is one of the largest trial on low-protein diet in CKD, the weight suddenly declined during the first months of LPD irrespectively of either the CKD stage or the amount of protein prescription and thereafter remained stable during the follow-up [39]. In that study the energy intake was low in all the sub-groups during the LPD while the weight decline was observed only at start; if the low energy or proteins had an adverse impact on nutrition, it would be observed a similar decline up to the end of the study and, therefore, other factors contributed to the weight loss. A possible explanation is that in CKD the protein intake is strictly parallel to sodium intake [40]; in CKD, that is a salt-sensitivity condition, a sudden decline of sodium intake associated to the low-protein diet reduces the extracellular volume [41]. As a result, the sudden weight loss after LPD start may be due to water loss. In this study, the body weight decline at start was associated with a slight reduction of body water which can explain at least a portion of the weight loss.

A further change in body composition detected along the study was a small decline of fat mass. Fat free mass decreased by the same extent in both groups but such a decline may be attributed to the loss of body water; indeed, the fractional FFM did not reduce during the diet. Conversely, the muscle functionality evaluated by the hand-grip strength improved in DM but not non-DM during the follow-up on LPD-KA. Interestingly, sarcopenia defined by handgrip strength and skeletal muscle mass estimated by BIA has been shown as an independent predictor of mortality in CKD [42]; according to a threshold for hand-grip strength, in this study sarcopenia estimated by muscle strength was highly prevalent at baseline and slightly improved during the study [21].

Given the observational design of the study and the slight differences between groups at baseline, we cannot exclude that unmeasured confounders and diabetic status could have influenced the nutritional effect of LPD-KA diet. However, several multivariable analyses strongly suggest that diabetes seems not to play a role on the nutritional effects observed during the long-term observational study.

To obtain a comprehensive evaluation of PEW by the combination of several nutritional parameters, we analysed together one parameter for each of the main categories suggested by the International Society of Renal Nutrition and Metabolism [6]. Low levels of albumin and BMI were present in around one over three patients at start and this percentage did not change during the study; low cholesterol was absent in all patients at any time; prevalence of reduced fat mass was very low at start and did not worsen in both groups during the study period. Overall, the nutritional status and body composition according these combined criteria, did not worsen in diabetics CKD while on LPD-KA. Few data are available from literature. In a systematic review of randomised controlled trials, serum albumin did not worse in CKD diabetics during LPD; the heterogeneity between trials, however, was high [26]. In contrast, in a small controlled trial in diabetic patients, serum albumin decreased during LPD but not in control diet; patients on LPD, however, decreased the energy intake along the study [24]. Therefore, the adherence to energy prescription is mandatory to avoid a negative impact of LPD on nutrition in diabetic CKD. This latter point has been clearly underlined in recent nutritional recommendations for CKD [38].

## Conclusion

This study provides first time evidence that in diabetic patient with advanced CKD a low-protein diet supplemented with ketoacids under a skilled dietitian supervision is safe. The major strengths of this study are that the nutritional status of the patients was comprehensively studied by a number of common either biochemical and clinical criteria or other tools for assessment of protein-energy wasting in CKD and that diabetic patients were compared along time with non-diabetics patients on the same intervention. A limitations of the study is that this is an observational but not randomized trial. Nonetheless, multivariate analyses does not disclose any association of diabetic status and main individual characteristics suggesting that the long-term effect of the low-protein diet is the same in diabetics and non-diabetics CKD patients. Therefore, the study concludes that a low-protein dietary supplemented with ketoacids reduces the accumulation of protein wasting products and ameliorates the glucose control improving the insulin sensitivity in diabetic CKD patients; furthermore, the study undoubtedly demonstrates that such restricted diet in the long term does not worsen the nutritional status and preserves the body composition avoiding the onset of sarcopenia in diabetic patients with CKD.

## Abbreviations

ANOVA: ANalysis Of Variance
BIA: BioImpedance Analysis
BMI: Body Mass Index
BMR: Basal Metabolic Rate
BW: Body Weight
CKD: Chronic Kidney Disease
CKD-EPI: Chronic Kidney Disease Epidemiology
DBP: Diastolic Blood Pressure
DM: Diabetes Mellitus
eGFR: estimated Glomerular Filtration Rate
ESRD: End Stage Renal Disease
FFM: Fat-Free Mass
FM: Fat Mass
GFR: Glomerular Filtration Rate
iBW: ideal Body Weight
KDIGO: Kidney Disease Improving Global Outcomes
LPD: Low-Protein Diet
LPD-KA: Low-Protein Diet plus KetoAcids
LPDs: Low-Protein Diets
MDRD: Modification of Diet in Renal Disease
MFES: Mexican Food Equivalent System
non-DM: non-Diabetes Mellitus
NS: Non Significant
NYHA: New York Hart Association
PEW: Protein-Energy Wasting
SBP: Systolic Blood Pressure
SD: Standard Deviation
TBW: Ttotal Body Water
W/H: Waist/Hip
